# Carotid Plaque Morphology is Similar in Patients with Reduced and Normal Renal Function

**DOI:** 10.1177/1179546820951793

**Published:** 2020-08-25

**Authors:** Caroline Heijl, Fredrik Kahn, Andreas Edsfeldt, Christoffer Tengryd, Jan Nilsson, Isabel Goncalves

**Affiliations:** 1 Department of Cardiology, Clinical Sciences, Lund University, Lund, Sweden; 2 Department of Infection Medicine, Clinical Sciences, Lund University, Lund, Sweden; 3 Experimental Cardiovascular Research Unit, Clinical Sciences, Lund University, Malmö, Sweden

**Keywords:** Atherosclerosis, chronic kidney disease, cardiovascular disease, stroke

## Abstract

**Background:**

Chronic Kidney Disease (CKD) is associated with an increased risk for cardiovascular events such as stroke. However, it is still unclear if decreased kidney function is associated with a vulnerable atherosclerotic plaque phenotype. To explore if renal function was associated with carotid plaque vulnerability we analyzed carotid plaques obtained at surgery from the Carotid Plaque Imaging Project (CPIP).

**Methods:**

Patients were enrolled through the CPIP cohort. The indication for surgery was plaques with stenosis >70%, associated with ipsilateral symptoms or plaques with stenosis >80% not associated with symptoms. Transversal sections from the most stenotic plaque region were analyzed for connective tissue, calcium, lipids, macrophages, intraplaque hemorrhage, and smooth muscle cells. Homogenates were analyzed for collagen and elastin.

**Results:**

Carotid endarterectomy specimens from 379 patients were obtained. The median GFR was 73 ml/min/1.73 m^2^. Plaque characteristics showed no significant association with eGFR, neither when eGFR was divided in CKD groups nor when eGFR was handled as a continuous variable and adjusting for other known risk factors (ie, age, diabetes, hypertension, and smoking).

**Conclusions:**

The higher risk of cardiovascular disease such as stroke in CKD is not associated with increased plaque vulnerability and other factors have to be sought.

## Introduction

Chronic renal disease (CKD) and atherosclerosis are closely associated, presumably due to both the renal disease itself, as well as common risk factors such as smoking, diabetes, and hypertension. CKD is a well-recognized risk factor for the development of cardiovascular disease (CVD) such as stroke, myocardial infarction, and heart failure.^1-3^ A linear relationship between decreasing estimated glomerular filtration (eGFR) and the risk of stroke has been described in a large meta-analysis,^4^ with a relative risk increase of 7% for every 10 mL/min/1.73 m^2^ reduction in eGFR below 90 ml/min/1.73 m^2^. Cardiovascular risk in end stage renal disease (ESRD), especially patients receiving hemodialysis, is more extensively studied than in patients with less severely reduced kidney function.^5^ Media calcification with increased arterial stiffness is a known risk factor in the dialysis population but the intima has to our knowledge been less studied.^6^ There are at present few and somewhat contradictory data regarding CKD and carotid plaque composition where both enhanced calcification, reduced collagen, and intraplaque hemorrhage has been described with diverging results in 2 studies.^7,8^ At present it remains unclear if a decreased kidney function is associated with the vulnerable plaque phenotype.^7,8^ A vulnerable atherosclerotic plaque has a large lipid core, covered by a thin fibrous cap, rich in inflammatory infiltrates, and possibly intraplaque hemorrhage.^9,10^ In this study we aimed to explore if the degree of renal function was associated to a vulnerable plaque phenotype in the Carotid Plaque Imaging Project (CPIP) cohort, that includes symptomatic and asymptomatic patients undergoing carotid endarterectomy.^11^

## The Carotid Plaque Imaging Project cohort

Patients were enrolled through the CIPIP cohort, which has been described elsewhere.^12^ In summary, carotid plaques were collected at carotid endarterectomies where indication for surgery were plaques with stenosis >70%, associated with ipsilateral symptoms (transient ischemic attack, stroke, or amarosis fugax) or plaques >80% not associated with symptoms. The extent of plaque stenosis was measured with duplex. All patients were preoperatively assessed by a neurologist. Informed consent was given from each patient and the study was approved by the local ethics committee (Regionala Etikprövningsnämnden i Lund, reference number 472/2005).

## Sample preparation and histology

After surgical removal, plaques were snap-frozen in liquid nitrogen in the operating room. Plaques were weighed and homogenates were prepared as previously described.^12^ Fragments of 1 mm, from the most stenotic region, were taken for histology/immunohistochemistry. Transversal sections from the fragment were analyzed by histology for evaluation of connective tissue (Masson trichrome), lipid content (Oil Red O) and calcium (Von Kossa) and by immunohistochemistry for evaluation of macrophages (CD68), intraplaque hemorrhage (glycophorin A) and smooth muscle cells (alpha-actin). The rest of the plaques was homogenized and analyzed by colourimetric assay of for assessment of collagen and elastin.

## Vulnerability index

To evaluate the combined importance of analyzed plaque components for vulnerability, a vulnerability index was used. The plaque vulnerability index was based on the sum of the percent plaque areas stained for lipids, macrophages, and hemorrhage, divided with the sum of the percent plaque areas stained for smooth muscle cells and collagen.

## Kidney function estimation

For all patients, relative estimated glomerular function (eGFR) was calculated using the LM-LBM revised^13^ and the eGFR was related to body surface area and expressed in ml/min/1.73 m^2^. The kidney function was used throughout the analyses either as continuous variables or divided in CKD-groups.

## Clinical outcome

All patients were followed up and adverse cardiovascular events such as myocardial infarction, stroke, transient ischemic attack (TIA), amaurosis fugax, and any vascular intervention not planned at the time of the operation such as endarterectomy or carotid artery stent, coronary artery bypass grafting or percutaneous coronary artery intervention, and any death by cardiovascular diseases.

Cardiovascular events and causes of death were assessed from the Swedish National Board of Health and Welfare (Socialstyrelsen) with data from the Swedish national in-patient health and the Swedish cause of death register together with telephone interviews with the patients and revision of medical charts. The ICD codes used to identify CV events were G45.9, G45.3, G46, I63.1-5, I63.8-9, I64.5, I21-22, I24.8-9, I25.1-2, I25.5-6, and I25.8. In this study the following ICD-10 codes were used to define cardiovascular death; I.10, I.13.2, I20.9, I21.9, I25.1, I.25.5, I25.8-9, I46.9, I48.9, I50.9, I60.9, I61.9, I63.9, I64.9, I69.4, I71.0, I73.9, and I74.9.

## Statistical methods

Means, medians, standard deviations (SDs), and interquartile ranges (IQRs) were reported as appropriate. Differences in frequencies between groups were tested with the chi^2^-test and Fisher’s exact test as appropriate and differences between group medians with the Mann-Whitney U test and Kruskal-Wallis test. Semi-partial correlation was used to assess the association between risk factors and plaques composition. Furthermore, an additive model using thin plate splines with smoking, hypertension, gender and diabetes as factors and age and eGFR as continuous variables was used to asses plaque composition. For statistical analyzes the eGFR was centered around 60 ml/min/1.73 m^2^ and divided by 10. Age was centered around 70 years and expressed as decades. Before using the additive model, the distribution of respective plaque content was sought and the additive model was adjusted concurrently. Next, the additive model including all variables was fitted and the *P*-value of each variable was noted. Since there was a suspicion that age and eGFR may show collinearity the concurvity between age and eGFR was investigated. The full model was compared to a model not including eGFR as an explaining variable. For this comparison both the Akaike Information Criterion (AIC) and an F-test comparing the full model versus the model lacking eGFR was performed. When comparing models, the maximum likelihood (ML) was used and when investigating the importance of explaining variables the restricted maximum likelihood (REML) was used. To investigate major adverse cardiovascular events (MACE) a cumulative incidence plot was made with death due to other causes as a competing event. The difference in incidence curves between different eGFR-groups were assessed using Gray’s test. To isolate factors important for CV events, a Cox-proportional hazard model was fitted using smoking, hypertension, gender and diabetes as dichotomous factors and age and eGFR as continuous variables. In this model, death due to other causes was treated as a censored event and hence the cause-specific hazard ratios were calculated. To verify the assumptions for the Cox-model, a Kaplan-Meyer plot was made and the differences between eGFR-groups were tested using the log-rank test. R (R Core Team [2017] R: A language and environment for statistical computing. R Foundation for Statistical Computing, Vienna, Austria. URL https://www.R-project.org/) was used for statistical computation with the following packages: readxl, haven, ggplot2, grid, ppcor, mgcv, fitdistrplus, and Hmisc.

*P*-values <.05 were regarded as significant.

## Results

### Patient characteristics

Carotid endarterectomy specimens from 379 patients were obtained, as previously described.^12^ The median follow- up period was 257.7 weeks (IQR 141.6-422.2). The renal function was estimated at baseline with the LM-LBM revised formula, the median GFR was 73 ml/min/1.73 m^2^ (range 18-144).^13^ Patients with lower eGFR were significantly older and had more frequently hypertension than patients with higher eGFR. Patients with eGFR <45 ml/min/1.73 m^2^ tended to have less diabetes and smoking was more common in the group without CKD, even if not statistically significant (Table 1).

**Table 1.** Baseline characteristics and carotid plaque composition in patients with normal kidney function, with mild reduction in kidney function (eGFR >60 ml/min/1.73 m^2^), with mild to moderate reduction (eGFR 45-60 ml/min/1.73 m^2^) and with moderate to severe reduction (eGFR <45 ml/min/1.73 m^2^). Unadjusted data.

	Combined	>60 ml/min/1.73 m^2^	45-60 ml/min/1.73 m^2^	<45 ml/min/1.73 m^2^	*P*-value
	n = 379	n = 281	n = 62	n = 36	
Age (years)					<.001^b^
• ⩽50, n (%)	7 (2%)	7 (2%)	0 (0%)	0 (0%)	–
• >50-60, n (%)	37 (10%)	37 (13%)	0 (0%)	0 (0%)	–
• >60-70, n (%)	138 (36%)	115 (41%)	20 (32%)	3 (8%)	–
• >70-80, n (%)	146 (39%)	103 (37%)	30 (48%)	13 (36%)	–
• >80-90, n (%)	51 (13%)	19 (7%)	12 (19%)	20 (56%)	–
Females, n (%)	124 (33%)	86 (31%)	22 (35%)	16 (44%)	.22^a^
Hypertension, n (%)	285 (75%)	199 (71%)	53 (85%)	33 (92%)	.0021^a^
Diabetes, n (%)	118 (31%)	87 (31%)	22 (35%)	9 (25%)	.59^a^
Smoking, n (%)	307 (81%)	235 (84%)	45 (73%)	27 (75%)	.081^a^
Oil Red O (% area)	n = 368	27 (16-37)	28 (15-37)	24 (16-35)	.93^b^
CD68 (% area)	n = 301	23 (15-34)	24 (15-34)	21 (14-35)	.49^b^
Glycophorin A (% area)	n = 241	5.2 (2.0-10.2)	4.7 (2.1-10.2)	5.5 (1.4-9.5)	.86^b^
Alpha-actin (% area)	n = 304	20 (13-30)	21 (14-31)	17 (13-27)	.09^b^
Van Kossa (% area)	n = 203	2.50 (0.81-6.19)	2.50 (0.88-5.76)	4.14 (0.92-7.25)	.11^b^
Vulnerability index	n = 197	1.9 (1.1-3.3)	2.2 (1.1-3.6)	1.8 (1.3-3.0)	.45^b^
Elastin (mg/g)	n = 219	52 (35-82)	53 (35-87)	52 (36-69)	.73^b^
Collagen (mg/g)	n = 230	47 (32-68)	50 (32-71)	40 (29-59)	.21^b^

eGFR, estimated glomerular filtration; CD, cluster of differentiation; AIC, Akaike Information Criterion.

Differences in frequencies between eGFR groups were tested with the chi^2^-test and Fisher’s exact test as appropriate and differences between group medians with the Mann-Whitney U test (^a^) and Kruskal-Wallis test (^b^).

### Plaque characteristics

Plaque characteristics showed no significant association with eGFR, neither when eGFR was divided in CKD groups^14^ (ie, G1-G2, G3a, and 3b-5 respectively) (Table 1) nor when eGFR was handled as a continuous variable and adjusting for other known risk factors in a semi partial correlation analysis (Table 2). To address the possibility of non-monotonous relationship between eGFR and plaque characteristics an additive model was constructed. After adjustment for known risk factors, eGFR had no significant predictive value on analyzed plaque components (Table 3).

**Table 2.** Association between risk factors and plaque composition.

Association between risk factors and plaque composition.	eGFR	Age	Gender	Diabetes	Smoking	Hypertension
Oil Red O (% area)	.69	.09	.26	.13	.30	.83
CD68 (% area)	.92	.28	.65	.01	.09	.38
Alpha actin (% area)	.23	.004	28	.01	.48	.20
Glycophorin A (% area)	.34	.05	.002	.07	.60	.37
Van Kossa (% area)	.22	.70	.04	.001	.42	.39
Masson (% area)	.22	.94	.11	.17	.83	.14
Vulnerability index	.37	.048	.29	.32	.29	.018
Elastin (mg/g)	.98	.03	.00	.003	.74	.38
Collagen (mg/g)	.40	.55	.001	.0002	.86	.57

eGFR, estimated glomerular filtration; CD, cluster of differentiation.

To adjust for confounders a semi-partial correlation with Kendall was used to assess the association between risk factors and plaque composition. In each column, *P*-values for the different predictors are displayed.

**Table 3.** Association between risk factors and plaque composition analyzed with a generalized additive model.

	eGFR	Age	Gender	Diabetes	Smoking	Hyper-tension	AIC full model	AIC excluding eGFR	F-test ***P***-values
Oil Red O (% area)	.83	.1	.51	.23	.44	.90	3001	2999	.79
CD68 (% area)	.32	.45	.66	.04	.17	.37	2426	2424	.91
Alpha actin (% area)	.48	.01	.22	.21	.58	.17	2362	2360	.52
Glycophorin A (% area)	.09	.16	.01	.16	.08	.74	1434	1434	.12
Van Kossa (% area)	.32	.85	.095	.002	.47	.13	972	971	.31
Masson (% area)	.15	.38	.39	.14	.85	.44	2444	2444	.15
Vulnerability index	.41	.18	.82	.16	.65	.15	733	733	.30
Elastin (mg/g)	.33	.003	.002	.02	.31	.93	2186	2185	.35
Collagen (mg/g)	.99	.27	.002	.001	.68	.76	2150	2148	.98

eGFR, estimated glomerular filtration; CD, cluster of differentiation; AIC, Akaike Information Criterion.

To verify the results in Table 2, a generalized additive model with thin plate splines was fitted with all predictors and the assumptions were verified. The AIC of the full model (including eGFR) was compared with the model without eGFR both with AIC as well as with an F-test. When comparing the models, the maximum likelihood was used. The *P*-values for the different predictors were then calculated and the restricted maximum likelihood was used.

### Clinical outcome

**Figure 1.**
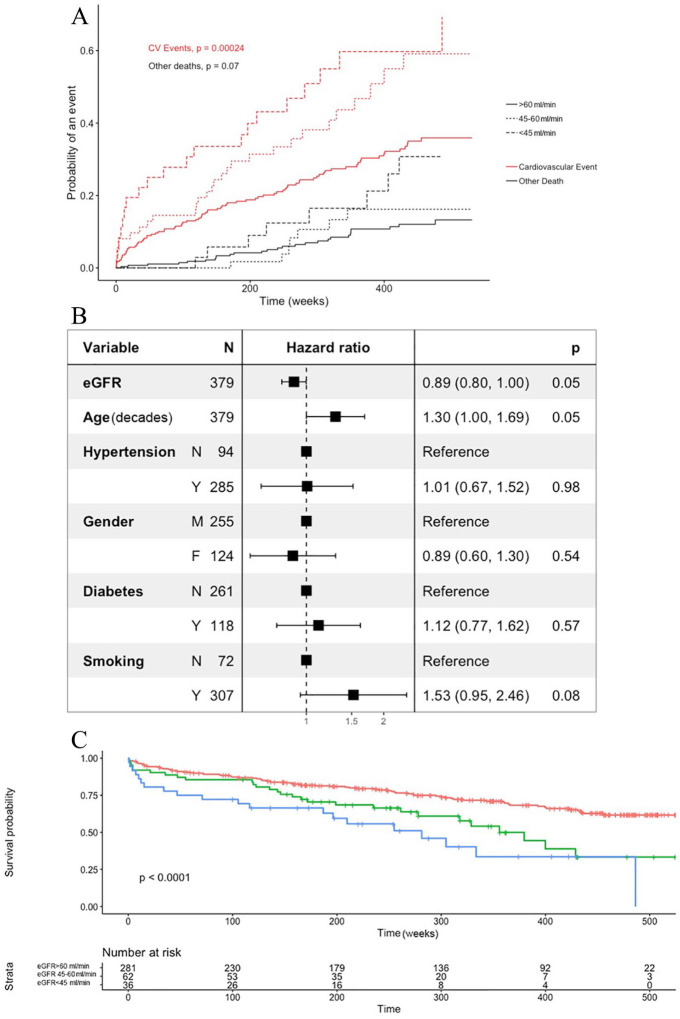
Clinical outcome. (A) To investigate cardiovascular events a cumulative incidence plot was made with death due to other causes as a competing event. The difference in incidence curves between different eGFR-groups were assessed using Gray’s test (supl). (B) To isolate factors important for CV events, a Cox-proportional hazard model was fitted using smoking, hypertension, gender and diabetes as factors and age and eGFR as continuous variables expressed as decades. In this model, death due to other causes was treated as censored events and hence the cause-specific hazard ratios were calculated. (C) Hazard curves for cardiovascular death after carotid endarterectomy. Death due to other causes was treated as censored events. The median follow- up period was 257.7 weeks (IQR 141.6-422.2). CV events, cardiovascular events; eGFR, estimated glomerular filtration.

There was a significant difference in the cumulative incidence of CV events between the 3 CKD groups (Figure 1A) when treating death due to other causes as a competing event. In a Cox-proportional hazard model where the cause-specific hazards were investigated (Figure 1B), eGFR was only borderline significant (*P* = .05) for CV events. Death due to other causes was treated as censored events. To further assess the possible confounding effect of age the cohort was divided in 3 equal sized groups based on age, giving cut-offs of 68 and 75 years, respectively. When assessing the effect of eGFR the effect seemed homogenous across all strata, although not always significant due to the lower number of patients in respective strata (Supplemental Figure 1).

## Discussion

In this study of a large cohort of patients going through carotid endarterectomy we show that renal function is not an independent risk factor for the vulnerable plaque phenotype. Other factors such as diabetes mellitus and hypertension are of significantly greater importance. This is in contrast to 2 other studies addressing a similar question. In 1 smaller cohort consisting of 114 patients, 51 with CKD and 63 without CKD (mean eGFR, 49 vs 88 mL/min/1.73 m^2^), Pelisek et al described enhanced calcification and reduced collagen in plaques from the group with mean eGFR of 49 ml/min/1.73 m^2^. However, these were crude data and no consideration to coexisting morbidities such as hypertension, age or diabetes were made. Wesseling et al however, conducted a well-designed study in their large cohort of 1796 patients undergoing CEA taking comorbidities into account. In this study the authors concluded that patients with reduced renal function did not have an association with inflammatory plaque characteristics, but on the other hand, an association was seen between poor renal function and intraplaque hemorrhage, as well as with all-cause mortality. One of the differences between these other cohorts and what we regard as strengths in ours, is that in CPIP all plaque data has continuous variables whether in the Athero-Express Biobank used by Wesseling et al many variables were only in semi quantitative or qualitative binominal groups. Moreover, in our study we address the statistical analyses without presupposing a linear relationship. It is clear in the work by Wesseling et al, that several plaque and plasma parameters do not exhibit a linear relationship with renal function and therefore analyses that require this as an assumption may not be utterly correct. We have chosen to evaluate both renal function and plaque phenotype data as continuous variables enabling us to detect any variation. In addition, the use of a combined plaque phenotype parameter—the vulnerability index—adds another strength as 1 plaque characteristic often interact or colocalizes with others within the same plaque.

A weakness with this study, as in other CEA cohorts is that there is some selection bias as patients accepted for CEA have to be considered fit enough to undergo an operation balancing the peri-operative risks. This might for example, explain why diabetes mellitus is less common in the patients with the most severely impaired renal function in this cohort compared with the general CKD-population. It should also be considered that all patients accepted for surgery have an advanced atherosclerotic disease. Therefore, if a reduced renal function is associated to a specific plaque morphology in less advanced plaque could not be explored in the present study. Finally, the information about smoking status in the patients are displayed in 2 groups with 1 group consisting of those who have never smoked and 1 group of both current and previous smokers. The cohort is too small to divide in 3 groups that would otherwise have been appropriate and this may overestimate the impact of smoking on plaque phenotype.

The question raised and not fully answered neither by our results, nor by the previous studies mentioned above, is if the carotid plaque phenotype in patients with CKD can explain the higher risk for cardiovascular disease seen epidemiologically in this group. Calcification, that would be expected to play a role in CKD patients with disturbed mineral-bone-axis, does not seem to have any significant importance when taking age into account. Inflammation, known to play a role in at least patients in dialysis, does not seem to be significantly different in plaques from patients with mildly to moderately impaired kidney function. Hemorrhage, according to Wesseling et al may play a role but the impact in their study was low with a HR of 1.2 and would probably not explain the markedly increased risk for cardiovascular events seen in patients with CKD.

In conclusion, we show that renal function does not seem to have an impact on known plaque characteristics defining a vulnerable or rupture-prone carotid plaque phenotype in patients undergoing CEA. Further studies are needed to find an explanation to why patients with renal disease have a higher risk for stroke and other cardiovascular diseases.

## Supplemental Material

Supplemented_figure_1 – Supplemental material for Carotid Plaque Morphology is Similar in Patients with Reduced and Normal Renal Function Click here for additional data file. Supplemental material, Supplemented_figure_1 for Carotid Plaque Morphology is Similar in Patients with Reduced and Normal Renal Function by Caroline Heijl, Fredrik Kahn, Andreas Edsfeldt, Christoffer Tengryd, Jan Nilsson and Isabel Goncalves in Clinical Medicine Insights: Cardiology
