# Supplementary material for: Carotid Plaque Morphology is Similar in Patients with Reduced and Normal Renal Function
Source: Clin Med Insights Cardiol. 2020 Aug 25;14:1179546820951793. doi: 10.1177/1179546820951793 (PMC7450288; doi:10.1177/1179546820951793)
Supplement: Supplemented_figure_1 – Supplemental material for Carotid Plaque Morphology is Similar in Patients with Reduced and Normal Renal Function [file Supplemented_figure_1.docx]

Supplemented figure 1. Clinical outcome in different strata based on age.


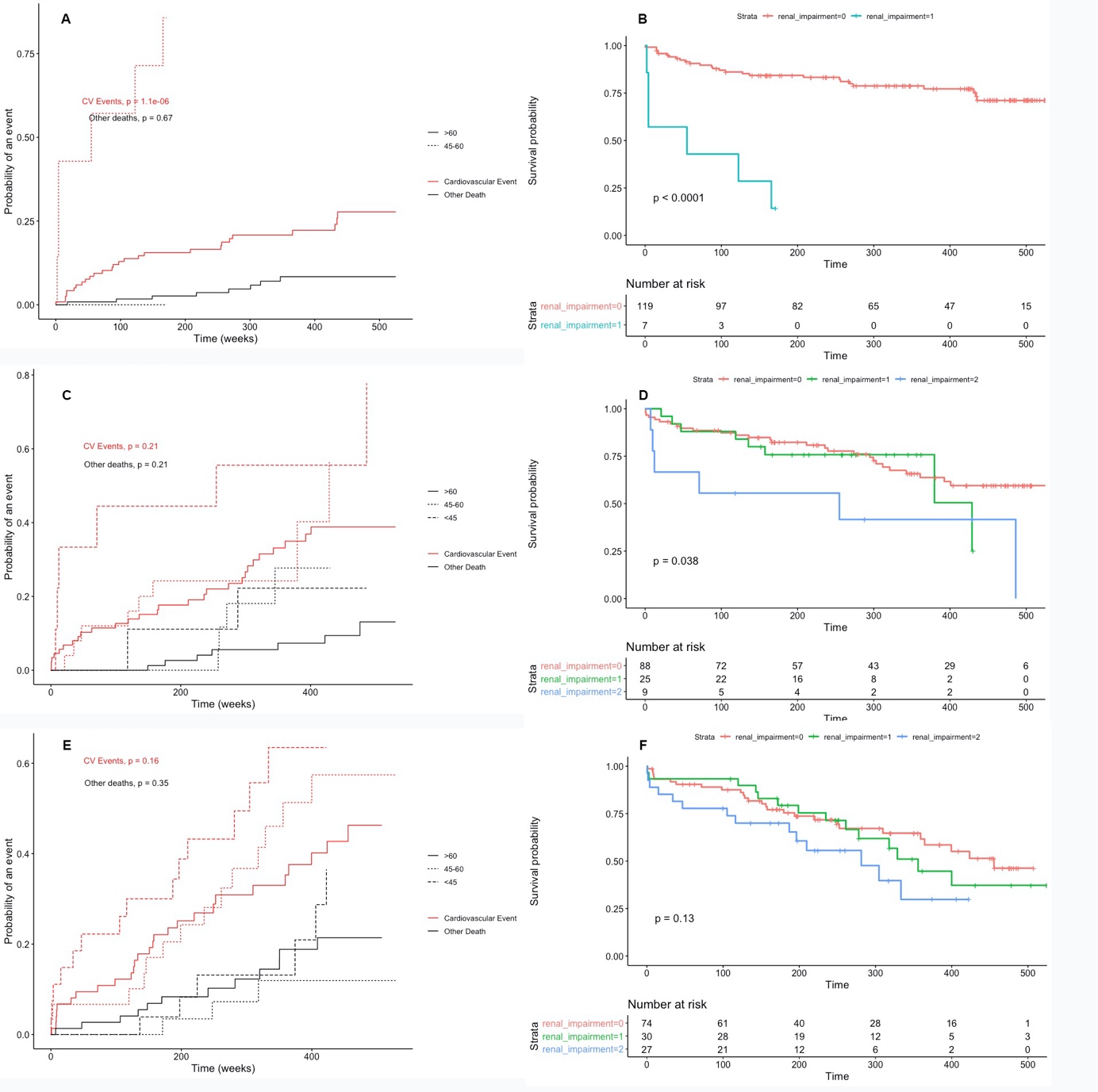


Supplemented figure 1. The cohort was divided in three equal sized strata based on age. Since age was entered as an integer the actual number of patients in the different strata were 126, 122 and 133 patients, respectively. The cut-offs for age were 68 and 75 years, respectively.

To investigate cardiovascular events a cumulative incidence plot was made with death due to other causes as a competing event. The difference in incidence curves between different eGFR-groups were assessed using Gray’s test. Furthermore, hazard curves for cardiovascular events after carotid endarterectomy were also made where death due to other causes was treated as a censored event. A) Cumulative incidence plot for patients <68 years. B) Kaplan-Meyer-plot for patients <68 years. C) Cumulative incidence plot for patients ≥68 years and <75 years. D) Kaplan-Meyer-plot for patients ≥68 years and <75 years. E) Cumulative incidence plot for patients ≥75 years. F) Kaplan-Meyer-plot for patients ≥75 years.
